# Effect of Bmi1 over-expression on gene expression in adult and embryonic murine neural stem cells

**DOI:** 10.1038/s41598-018-25921-8

**Published:** 2018-05-10

**Authors:** Mythily Ganapathi, Nathan C. Boles, Carol Charniga, Steven Lotz, Melissa Campbell, Sally Temple, Randall H. Morse

**Affiliations:** 1 Laboratory of Molecular Genetics, Wadsworth Center, New York State Dept. of Health, Albany, NY USA; 2 Neural Stem Cell Institute, Rensselaer, NY USA; 3 Department of Biomedical Science, University at Albany School of Public Health, Albany, NY USA

## Abstract

The ability of isolated neural stem cells (NSCs) to proliferate as neurospheres is indicative of their competence as stem cells, and depends critically on the polycomb group (PcG) member Bmi1: knockdown of *Bmi1* results in defective proliferation and self-renewal of isolated NSCs, whereas overexpression of *Bmi1* enhances these properties. Here we report genome-wide changes in gene expression in embryonic and adult NSCs (eNSCs and aNSCs) caused by overexpression of Bmi1. We find that genes whose expression is altered by perturbations in Bmi1 levels in NSCs are mostly distinct from those affected in other multipotent stem/progenitor cells, such as those from liver and lung, aside from a small core of common targets that is enriched for genes associated with cell migration and mobility. We also show that genes differing in expression between prospectively isolated quiescent and activated NSCs are not affected by Bmi1 overexpression. In contrast, a comparison of genes showing altered expression upon Bmi1 overexpression in eNSCs and in aNSCs reveals considerable overlap, in spite of their different provenances in the brain and their differing developmental programs.

## Introduction

NSCs are maintained throughout embryogenesis in the developing mammalian cerebral cortex, where they give rise to neurons in deep and then more superficial cortical layers, and then switch to producing glial cells^1,2^. In contrast to the changing developmental potential of eNSCs, adult NSCs, found in the subgranular zone of the hippocampal dentate gyrus and the subventricular zone (SVZ) of the lateral ventricles, continue to generate neurons throughout life^3^. Both adult and embryonic NSCs, when isolated from the mouse brain and grown as primary cultures under non-adherent culture conditions in the presence of mitogens, produce multicell spheres or ‘neurospheres’^4^. The self-renewal of both eNSCs and aNSCs can be readily demonstrated by passaging neurospheres multiple times, with maintenance of expression markers including LeX /SSEA-1^5^ and GFAP and demonstrated multipotency^6,7^.

The regulation of the self-renewal and differentiation capacity of NSCs is of great interest both from the standpoint of potential therapeutic applications and the understanding of development, maintenance, and repair of the central nervous system throughout life^8^. A critical regulator of NSC function that has emerged from recent studies is Bmi1^9–11^. Bmi1 is a member of the polycomb group complex, which plays a key role in controlling expression of developmental regulators in a variety of lineages in metazoans^12,13^. As a member of the PRC1 complex, Bmi1 cooperates with its PRC1 partner, Ring1B, to ubiquitylate Lysine119 of histone H2A, a key step in PcG-mediated gene repression^14^. Bmi1 has been implicated in regulating several varieties of somatic stem cells^9–13,15–17^. Knockdown of *Bmi1* using shRNA causes severe defects in NSC self-renewal and differentiation capacity, while overexpression of *Bmi1* enhances these properties both *in vitro* and *in vivo*^11^. Defects in self-renewal and proliferation observed upon *Bmi1* knockdown were found to be mediated by cell cycle inhibitors p16, p19, and p21^10,11,18,19^.

Overexpression of Bmi1 increases self-renewal and proliferation of NSCs both *in vitro* and *in vivo*^11^; however, changes in genome-wide expression that accompany Bmi1 overexpression have not been reported. Furthermore, although cortical eNSCs and aNSCs exhibit substantial similarities, they also differ in important regards. First, they occupy distinct niches in the brain. Second, eNSCs alter their developmental potential with time to accommodate a temporal program^2^, whereas aNSC potential is less broad and subject to niche-specific inputs that differ from those influencing eNSCs^20,21^. Importantly, overexpression of Bmi1 can prevent the loss of neurogenic potential seen in neurospheres derived from the aNSC zone, indicating that this treatment can help this lineage self-renew more effectively^11^. A direct comparison of the transcriptome of eNSCs and aNSCs, and of the effect of Bmi1 overexpression on their genome-wide transcription, could provide important clues regarding similarities and differences of these two varieties of NSCs and the nature of self-renewal.

To address these issues, we have measured the effect of Bmi1 overexpression on genome-wide transcription in eNSCs and aNSCs *in vitro*. The expression data we report will provide a useful resource for other investigators interested in NSC self-renewal and function during neurogenesis.

## Results and Discussion

### Genes downregulated by Bmi1 overexpression in aNSCs are enriched for genes involved in neurogenesis

The purpose of the present study was to identify regulatory targets of Bmi1 and to shed light on downstream effectors and pathways that contribute to the NSC phenotype. To this end, we used microarrays to compare gene expression in adult SVZ-derived, cultured NSCs overexpressing Bmi1 versus control aNSCs from the same preparations transduced with empty vector. Both control and Bmi1-overexpressing aNSCs were cultured under non-adherent conditions to allow neurosphere formation. Overexpression of Bmi1 varied from 4 to 24 fold (Supplementary Table S1), and neurospheres formed by cells over-expressing Bmi1 were much larger than in control populations, consistent with increased proliferation and enhanced survival of NSCs^6,11^ (Supplementary Fig. S1). Indeed, aNSCs overexpressing Bmi1 also showed increased proliferation (Supplementary Fig. S1C), and exhibited an increased capacity for differentiation into neurons (Supplementary Fig. S1E).

RNA was isolated one week after the third passage for four biological replicate experiments, and gene expression was measured using Affymetrix Mouse Genome 430 2.0 microarrays. Processing of this data using the Cistrome platform^22^ revealed 1208 genes (7.3% of those assayed, based on Entrez IDs) having altered expression with false discovery rate (FDR) <0.10 (Supplementary Data File 1). Analysis using Genespring, which employs a slightly different algorithm for combining intensity values for multiple probes into expression values for single genes, yielded similar results (data not shown). Results were validated by conducting qPCR analysis of selected down- and up-regulated genes, which was in general agreement with values obtained in the microarray analysis (Supplementary Fig. 2).

**Figure 1:**
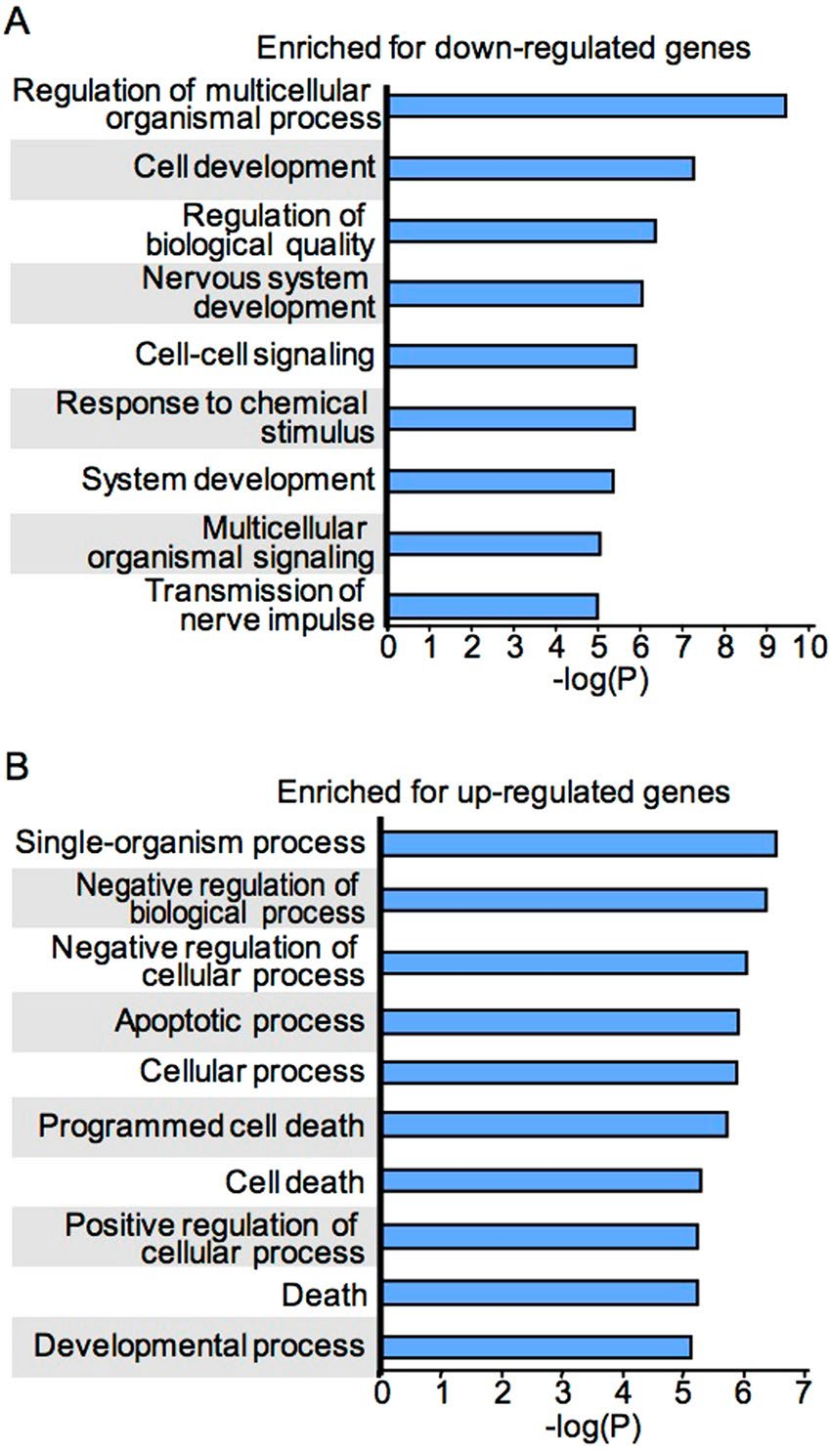
Gene ontology enrichment among genes affected by Bmi1 overexpression in aNSCs. Categories enriched for genes down-regulated (**A**) or up-regulated (**B**) at least two-fold upon Bmi1 overexpression in aNSCs are indicated along with −log of the corrected p-value for each category. All categories having corrected p-value <10^−5^ are shown except for unannotated genes, which were enriched among down-regulated genes.

A total of 904 genes were ≥2 fold differentially regulated between aNSCs overexpressing Bmi1 and control aNSCs transduced with empty vector (521 genes downregulated; 383 genes upregulated), indicating a modest tendency towards repression by Bmi1 overexpression. Gene ontology (GO) analysis of genes down-regulated at least two-fold by Bmi1 overexpression revealed enrichment of several terms related to neural development and differentiation (Fig. 1). Among 59 enriched GO terms with corrected p-value < 0.01, nine relate to neural development or function and 18 to development or differentiation (Supplementary Table S2); many of these terms contain overlapping sets of genes. This down-regulation is likely the reason for the restricted differentiation seen in Bmi1 overexpressing neural progenitors^11^, and may also contribute to the enhanced maintenance of stem cell characteristics in these neurospheres. For genes upregulated in Bmi1-overexpressing NSCs, the strongest enrichment was seen in high-level categories such as regulation of cellular process and in terms related to apoptosis (Fig. 1B; Supplementary Table S3). Enrichment for genes in GO terms related to apoptosis was also found among down-regulated genes, albeit to lesser extent (Supplementary Table S2). These effects on genes functioning in apoptosis may be due in part to the strong down-regulation of *mir29b* caused by Bmi1 overexpression, as this microRNA has been shown to inhibit apoptosis during neuronal maturation^23^. *Bmi1* overexpression has been found to lead to increased apoptosis in embryonic cortical neural progenitors upon differentiation into neuronal lineages both *in vivo* and *in vitro*, mediated by specific repression of *Survivin*^11,24^. In contrast, Bmi1-overexpressing aNSCs are resistant to apoptosis^11^. Expression of *Survivin* is unaffected in our Bmi1-overexpressing aNSCs, suggesting that altered regulation of other genes involved in apoptosis may be insufficient to effect apoptotic programs under these circumstances.

Genes down-regulated by Bmi1 overexpression in aNSCs and known to be involved in neural regulatory processes include *Gfap*, *Bdnf*, *Nrn1*, *Neurod1*, *Twist1*, *Satb2*, and *En2* (Supplementary Table S2), while the most strongly up-regulated gene was *Gsx2*, constitutive expression of which has been reported to expand NSCs in the adult SVZ and block neurogenesis^25^. Many other strongly affected genes have not been previously implicated in neural stem cell regulation but may have novel roles that remain to be elucidated. For example, Prdm8, a putative H3K9 methyltransferase^26^, functions in neural circuit assembly and in timing events during neocortical development^27,28^, and has been implicated in development of upper-layer neocortical neurons^29^ as well as retinal bipolar cells^30^. Another down-regulated gene, Nap1l3, is the most strongly down-regulated of several histone-coding and nucleosome assembly factors, suggesting a role for nucleosome assembly in NSC regulation. Intriguingly, the histone chaperone CAF-1 was recently reported to be important in maintaining somatic cell identity, such that its suppression enhanced the efficiency of generating induced pluripotent stem cells from somatic cells^31^. Non-coding RNAs were neither significantly enriched nor depleted among down-regulated genes; 31 annotated RNA species (indicated by “NR” Refseq IDs) were present among 484 genes down-regulated by at least two-fold with FDR < 0.10, representing 6.4% of this cohort, compared to 5.4% of all genes queried by the micorarray.

Several cell cycle inhibitors have been previously characterized as targets of Bmi1-mediated repression, including p16 and p19, encoded by the Ink4a-Arf locus (*Cdkn2a*), and p21 (*Cdkn1a*)^10,11,18,19^. Our microarray data indicates that only p16/p19 differs substantially between Bmi1-overexpressing and control aNSCs (*Cdkn2a* down 3.2 fold; p = 0.004 (paired t-test)), though *Cdkn1a* showed slight down-regulation; this was confirmed by qPCR of independent biological replicates (Supplementary Fig. S2).

### Genes differing in expression between quiescent and activated NSCs are not affected by Bmi1 overexpression

**Figure 2:**
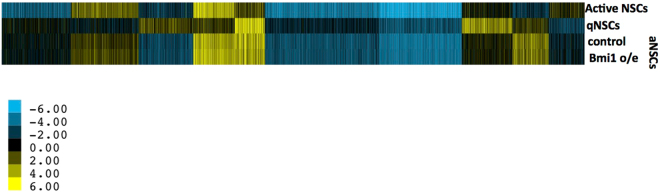
K-means clustering of gene expression in active and quiescent NSCs and aNSCs. K-means clustering (K = 10) of gene expression for active and quiescent aNSCs^32^, and for control and Bmi1-overexpressing aNSCs, was performed using genes that exhibited at least four-fold change in expression between active and quiescent adult NSCs. Expression values from microarray analyses were adjusted by subtraction of the mean value to allow values to center around zero. Genes belonging to the 10 clusters are listed in Supplementary Table S5.

Doetsch and coworkers have reported gene expression from prospectively isolated quiescent and activated aNSCs from the adult mouse brain, distinguished by lack or presence of EGFR, respectively, and concomitant proliferative activity^32^. Expression in prospectively isolated, activated aNSCs^32^ correlates well with the expression that we measured in aNSCs propagated as neurospheres (r^2^ = 0.63). Cluster analysis of those genes whose expression differs at least four-fold between quiescent and activated aNSCs shows that gene expression in aNSCs from our data using neurospheres is much more similar to that seen in activated than quiescent aNSCs (Fig. 2). Notably, genes differing in expression between quiescent and active aNSCs were not affected significantly by Bmi1 overexpression in this study, indicating that their differential regulation may be enforced by a mechanism independent of PcG-mediated regulation.

### Bmi-regulated expression in three adult multipotent cell types

**Figure 3:**
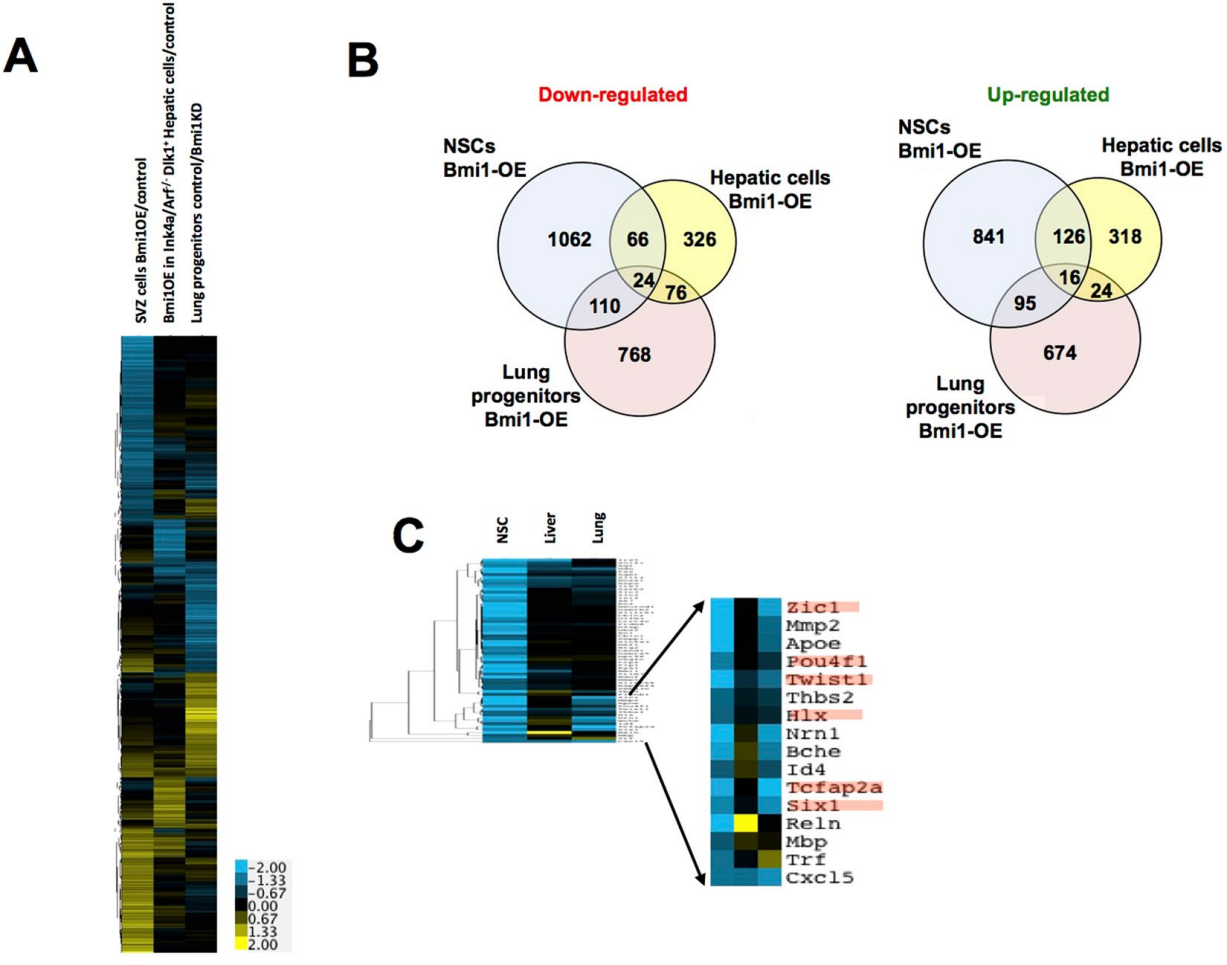
Effect of elevated Bmi1 expression in aNSCs, hepatic precursor cells, and lung stem cells. (**A**) Hierarchical clustering of 4202 genes showing altered expression of at least 1.5 fold in at least one of the three data sets indicated. (**B**) Overlap among genes up- or down-regulated by at least 1.5 fold in the indicated data sets. (**C**) Hierarchical clustering was performed on gene expression changes caused by elevated Bmi1 levels in aNSCs, hepatic precursor cells, and lung stem cells using 64 genes down-regulated at least 2 fold in aNSCs overexpressing Bmi1 and belonging to the GO category of nervous system development. The expanded region shows a cluster of genes most of which are also down-regulated in lung stem cells. Highlighted genes encode known and probable transcription factors.

The PcG complex has been implicated in repressing genes important for differentiation at early developmental stages of a number of different lineages^13^. This raises the question as to whether such regulation is primarily directed to common or distinct targets in different lineages. To address this question, we compared our results to two studies that investigated Bmi1 perturbation in other adult stem cell systems^17,33^. One of these studies compared expression of lung stem cells isolated from either wild type or a Bmi1 deficient mouse^17^. The second compared control and Bmi1 overexpressing Ink4a/Arf^−/−^ Dlk^+^ hepatic progenitor cells^33^, predicated on findings that much of the effect of Bmi1, including stem cell proliferation, has been ascribed to its repressive role on the cell cycle inhibitors Ink4a (p16Ink4a) – Arf (p19Arf) and p21^10^. Clustering analysis of all genes having expression altered by at least 1.5-fold in at least one of the datasets showed that the majority of genes affected in each stem cell type were not regulated by Bmi1 in the other two cell types (Fig. 3A). Overlap between datasets was nonetheless significant (Fig. 3B; all two-way p-values < 10^−13^ (hypergeometric test) except for up-regulated genes between lung stem cells and hepatic progenitors, p = 0.0036).

We asked whether those genes that were commonly up- or down-regulated among these datasets might reflect common functions needed for maintenance of the stem cell state or during differentiation, respectively. Genes that were down-regulated in the presence of higher levels of Bmi1 in aNSCs and either lung stem cells or hepatic progenitors were enriched in gene ontology (GO) categories involving blood vessel development and higher order processes associated with differentiation (e.g. anatomical structure morphogenesis, developmental process). We had expected to find categories associated with neurogenesis to be uniquely enriched among down-regulated genes in NSCs, but discovered that the GO terms were associated with genes down-regulated by higher Bmi1 levels in both aNSCs and lung stem cells (e.g. nervous system development, GO:0007399, corrected p-value of 5 × 10^−5^). Interestingly, commonly down-regulated genes from this category included several probable gene regulatory factors, suggesting their involvement in distinct differentiation pathways from the corresponding multipotent precursors (Fig. 3C, highlighted genes).

Examination of genes commonly down-regulated in all three cell types revealed an enrichment for genes associated with cell migration and mobility; eight of the 24 overlapping genes were associated with these categories (Ccl2, Ndn, Ccbe1, Slit2, Enpp2, Cxcl5, Foxc1, Nox4). Genes required for differentiating multipotent precursors to move away from their niche during differentiation may be commonly down-regulated by Bmi1 (and presumably PcG complex proteins) in a variety of contexts. Loss of such control could potentially contribute to carcinogenic phenotypes manifested by stem cells in particular PcG mutants.

Previous work identified imprinted genes as enriched targets of Bmi1-mediated repression in lung stem cells^17^. We did not observe similar enrichment for imprinted genes among those genes down-regulated by Bmi1 overexpression in aNSCs. Of 84 imprinted genes (http://mousebook.org/catalog.php?catalog=imprinting) that were interrogated in our microarray data, only seven were down-regulated by at least two-fold in aNSCs overexpressing Bmi1 (Supplementary Table S4). Thus, 8% of imprinted genes examined overlap with the 3.1% of genes that are down-regulated by Bmi1 overexpression, which is at most a modest enrichment (p = 0.011, hypergeometric test).

Among up-regulated genes, no significant gene ontology enrichment was observed between aNSCs and hepatic progenitors. Genes up-regulated in both aNSCs and lung stem cells overexpressing Bmi1 were enriched for associations with processes related to cell cycle, consistent with enhanced self-renewal. Taken together, comparison of Bmi1-regulated gene expression in three multipotent cell types indicates that Bmi1 governs regulatory programs that are largely specific to each cell type but that include a small core of genes associated with differentiation-related processes, and particularly with cell mobility and migration, which are common targets in distinct lineages.

### Bmi1 regulation of gene expression in embryonic compared to adult neural stem cells

Multipotent neural progenitor cells capable of giving rise to both neurons and glia are found in the embryonic as well as the adult mammalian brain. These eNSCs differ from aNSCs in that their developmental potential changes during embryogenesis, and they are therefore not self-renewing in the strictest sense^34^. They additionally differ from aNSCs in the physical niche they occupy; whereas aNSCs are found in the subgranular zone of the hippocampus and in the subventricular zone, cortical eNSCs reside in the anterior dorsal region where they progressively give rise to layers of the developing cortex^3,35^. The developmental potential of eNSCs is governed largely in a cell autonomous fashion, giving rise first to neurons and later to glial cells both during development and during prolonged culture *in vitro*^1,2^. Embryonic and adult NSCs also differ in their apoptotic response to Bmi1 overexpression in culture^11^. In spite of these differences, altered levels of Bmi1 similarly affect self-renewal potential in both cell types, as measured by neurosphere formation upon depletion or overexpression of Bmi1 in culture.

To determine the extent to which the transcriptional responses to Bmi1 overexpression are shared between eNSCs and aNSCs, we used RNA-seq to measure expression in isolated eNSCs (from E18 embryonic cortex) and aNSCs that were transduced in parallel either with control or Bmi1-overexpressing lentiviral vector immediately upon isolation. Transduced cells were then maintained as primary cultures under non-adherent conditions to allow neurosphere formation prior to RNA isolation. We obtained from each of two replicate samples 53–99 million mapped reads for Bmi1-overexpressing and control eNSCs, and from 13–59 million mapped reads for Bmi1-overexpressing and control aNSCs. Gene expression was analyzed and compared using Tophat and Cufflinks in the Galaxy server package^36^.

**Figure 4:**
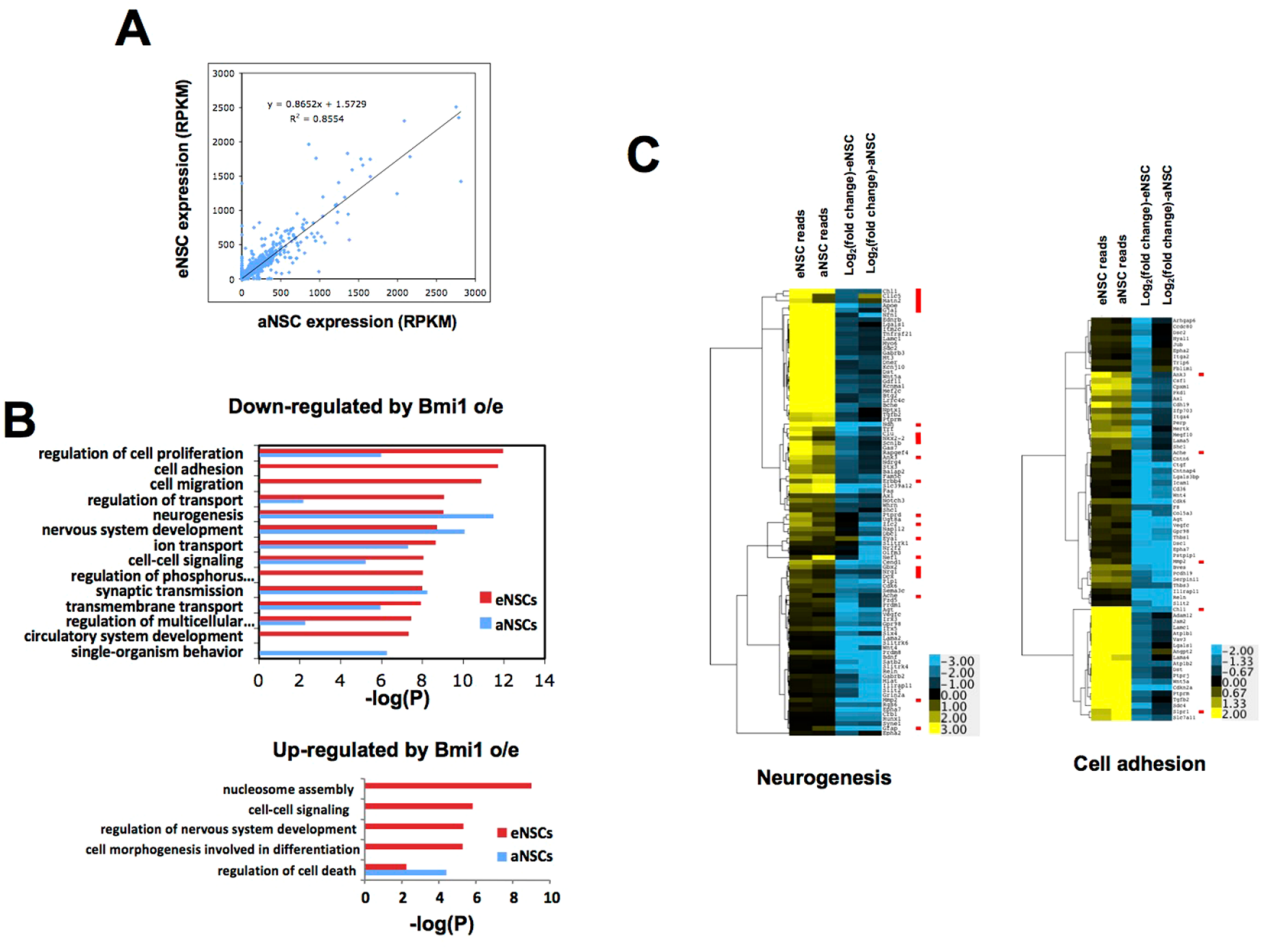
Effect on gene expression of Bmi1 overexpression in eNSCs compared to aNSCs. (**A**) Comparison of transcript levels measured by RNA-seq (reads per kilobase per million reads mapped, RPKM) in embryonic and adult NSCs (empty vector control, average of two biological replicates). (**B**) Gene ontology categories enriched for genes down-regulated (top) or up-regulated (bottom) upon Bmi1 overexpression in eNSCs (red bars) or aNSCs (blue bars). Categories having P < 10^−6^ (hypergeometric test, corrected for multiple category testing) are shown for down-regulated genes and having P < 10^−4^ for up-regulated genes; GO categories having more than 1624 member genes (the number in nervous system development) are not shown. (**C**) Hierarchical clustering was performed for genes down-regulated at least two-fold by Bmi1 overexpression with p < 0.01 in either eNSCs or aNSCs and belonging to the GO category Neurogenesis (left) or by at least two-fold in eNSCs with p < 0.01 and belonging to Cell Adhesion (right). The right-hand two lanes in each panel depict as heat maps log_2_ of the change in expression between cells overexpressing Bmi1 and control cells, while the left-hand lanes indicate relative expression levels in control cells (transduced with empty vector). Reads (RPKM) were divided by 6 prior to clustering to allow them to be visualized on the same scale as the log_2_ changes in expression. Red dots mark genes expressed at a level of at least 3 RPKM (before correction) and differing at least two-fold in expression level between eNSCs and aNSCs. Note that the color scales are different for the two clustering plots.

Gene expression was strongly correlated between eNSCs and aNSCs (Fig. 4A). We also compared expression changes caused by Bmi1 overexpression in aNSCs at one week and four weeks after isolation and transduction, and found moderately strong correlation (r^2^ = 0.61; Supplementary Fig. S3), indicating that a substantial portion of the expression changes observed correspond to relatively early (and therefore possibly direct) effects. To compare effects of Bmi1 overexpression in eNSCs and aNSCs, we first determined GO categories enriched for genes up- or down-regulated by Bmi1 overexpression in the two cell types (Fig. 4B). Both eNSCs and aNSCs showed enrichment among down-regulated genes for GO categories related to neural cell differentiation, such as neurogenesis and synaptic transmission. This is consistent with overexpression of Bmi1 maintaining both eNSCs and aNSCs in their undifferentiated state. Although several categories were enriched for eNSCs and not aNSCs or vice versa based on p-values (hypergeometric test), closer examination revealed that many genes in these categories showed similar responses to Bmi1 overexpression in eNSCs and aNSCs. For example, down-regulated genes in eNSCs were enriched in the category of cell adhesion based on 67 of 642 (10.4%) down-regulated genes belonging to this category, while only 3.4% of the total population belongs to this category (p < 10^−11^). Although aNSC down-regulated genes were not enriched in this category by our significance criterion, 32 of these same 67 genes were down-regulated at least two-fold. Thus, the difference in the behavior of cell adhesion genes in their response to Bmi1 overexpression in adult and embryonic NSCs is one of degree and not of kind. Additional examination of apparently differentially regulated GO categories showed this to be uniformly the case. Similarly, although genes up-regulated by Bmi1 overexpression in eNSCs were enriched in several categories that did not show equivalent enrichment in aNSCs (Fig. 4B), this again proved to be a quantitative, and not qualitative, distinction.

To investigate gene regulation in eNSCs compared to aNSCs more closely, we examined two GO categories that behaved differently with regard to enrichment in the two cell types. Neurogenesis showed enrichment for genes down-regulated by Bmi1 overexpression in both eNSCs and aNSCs, while cell adhesion was more significantly enriched for genes down-regulated in eNSCs (Fig. 4B). Comparison of the normalized number of reads between eNSCs and aNSCs for genes in both categories showed that their expression was generally similar in the two cell types (Fig. 4C, left-hand lanes). More genes involved in neurogenesis showed differential expression between eNSCs and aNSCs than did genes involved in cell adhesion (Fig. 4C, left-hand lanes; genes showing substantially different expression in eNSCs and aNSCs are marked with red dots; see also Supplementary Fig. S4). With regard to the effect of Bmi1 overexpression, similar numbers of genes involved in neurogenesis were down-regulated by Bmi1 in aNSCs and eNSCs, but many of these were affected more in one cell type than the other, and most affected genes were expressed at low to moderate levels; genes expressed at high levels tended to be least affected by increased Bmi1 expression (Fig. 4C; Supplementary Fig. S4). These results indicate broadly similar programs of gene expression and regulation in eNSCs and aNSCs, with these programs being differentially modulated to give rise to those differences observed here. The impact of the differential gene expression and differential regulation by Bmi1 between eSNCs and aNSCs for specific subsets of genes involved in neurogenesis, cell adhesion, and other gene ontology categories that are important for NSC function, and the mechanism underlying these subtle differential effects, will require further studies.

**Figure 5:**
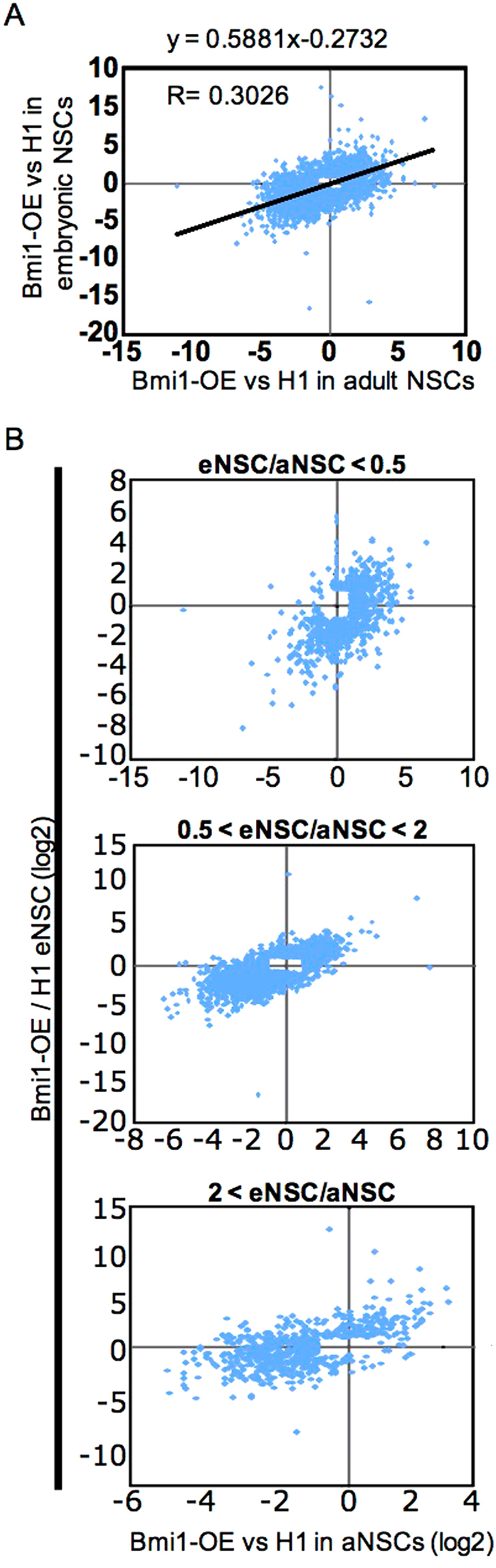
Comparison of gene expression changes in embryonic and adult NSCs caused by Bmi1 overexpression. (**A**) Reads per kilobase per million reads (RPKM) from RNA-seq data from aNSCs and eSNCs is plotted for 4296 genes having expression altered at least two-fold by Bmi1 overexpression in either eNSCs or aNSCs. (**B**) Genes plotted in (**A**) were broken down according to their relative expression in eNSCs and aNSCs as indicated.

To compare the effect of Bmi1 overexpression more broadly between eNSCs and aNSCs, we plotted the log_2_ of the change in expression elicited by Bmi1 overexpression in eNSCs against that for aNSCs for 4296 genes whose expression was changed at least two-fold by Bmi1 overexpression in at least one of the two cell types (Fig. 5A). A moderate (given the size of the data set) correlation was observed (r^2^ = 0.30; p < 10^−30^), indicating that genes are similarly regulated by Bmi1 in the two cell types. To gain additional insight, we replotted the data after segregating it into three sets depending on the ratio of expression in eNSCs and aNSCs (Fig. 5B). Genes having similar expression (within two-fold, based on RPKM) in eNSCs and aNSCs also showed generally similar effects of Bmi1 overexpression (Fig. 5B, middle plot). In contrast, genes expressed at lower levels in aNSCs than eNSCs were also more down-regulated by Bmi1 in aNSCs and vice versa (Fig. 5B, upper- and lower-most plots; Supplementary Fig. S5). This suggests that whatever induces higher expression of genes that are differentially expressed between eNSCs and aNSCs may cause those genes to become refractory to Bmi1-mediated repression. Although the mechanism responsible for this differential regulation is unclear, two possibilities are that Bmi1 (and presumably PcG) is differentially recruited to genes in eNSCs and aNSCs, or that other factors—possibly activators that increase transcription in one cell type over the other—override the repressive function of PcG at such genes preferentially in one of the two cell types. The latter mechanism would be consistent with various studies showing that repressive chromatin structures, both in yeast and metazoans, are more effective at suppressing weak than strong activators^37^.

## Conclusion

Overexpression of Bmi1 in NSCs from adult or embryonic mouse brain increases self-renewal and differentiation capacity both *in vivo* and *in vitro*^11^. These enhancements are due in part to down-regulation of the cell cycle inhibitors p16, p19, and p21^11^. However, the full range of genes affected by *Bmi1* overexpression had not been previously determined. Here we report genome-wide determination of gene expression in adult and embryonic NSCs, and the effect of Bmi1 overexpression on expression in both cell types.

We found similar patterns of gene expression in aNSCs and eNSCs when cultured as neurospheres, in spite of their distinct provenance and differentiation programs *in vivo*. It is possible that niche-specific, exogenous signals cause differential expression between eNSCs and aNSCs that is not seen during growth as neurospheres; this will be an interesting, if technically challenging, topic for future work.

Our results do not distinguish between genes that are direct targets of Bmi1 and those whose expression is indirectly affected. Chip-seq could help distinguish these, in future studies. The correlation observed between genes affected in aNSCs one week and four weeks post-isolation indicates that a substantial fraction of affected genes show altered expression at a relatively early time, implying that they do not reflect long-term effects of Bmi1 overexpression. The changes in gene expression that we report here, whether they are caused directly or indirectly by Bmi1 overexpression, allow comparison with gene expression programs in other lineages that are influenced by Bmi1 (presumably via the PcG complex), and also allow comparison between these programs in eNSCs and aNSCs.

Regarding the effect of Bmi1 overexpression, we observed a high degree of overlap among affected genes in adult and embryonic NSCs, which overlap very little with genes affected by altered Bmi1 expression in other (non-ectodermal) types of somatic stem cells. Genes down-regulated by Bmi1 overexpression in NSCs are enriched for GO terms related to proliferation, cell migration and mobility, neurogenesis, and apoptosis, four processes at the heart of neurogenesis^8^. Genes in these categories were mostly affected similarly by Bmi1 overexpression in adult and embryonic NSCs, though differences were observed. We also observed subsets of genes that were more strongly down-regulated by Bmi1 overexpression in aNSCs than in eNSCs or vice versa. Genes that were more strongly down-regulated in aNSCs were typically also expressed at lower levels in aNSCs than eNSCs having normal levels of Bmi1. This could suggest that such targets are selectively under Bmi1 regulation in aNSCs and not eNSCs (and conversely); whether by this or some other mechanism, determination of how differential regulation occurs at some targets awaits further research.

In summary, our results indicate regulation by Bmi1 of specific targets whose functions are intimately connected with the control of proliferation and neurogenic function in NSCs in both the developing and adult brain. How these targets are determined and the roles they play in neurogenesis will be important topics for future study.

## Materials and Methods

### Bmi1 overexpression vector

A Bmi1 clone was obtained from OpenBiosystems and cloned into a modified lentiviral vector driving expression from a Ubiquitin-C promoter^38^; the vector was modified by addition of an IRES eGFP to allow visualization of transduced cells^39^. An empty vector expressing only GFP was used as a control^39,40^.

### Neurosphere culture

For preparation of adult NSCs, SVZ tissue was dissociated from adult male Swiss Webster mice and cells were isolated as described previously^1^. Euthanasia of animals for preparation of NSCs was performed by pentobarbital overdose or CO_2_ inhalation. These methods are in accordance with recommendations of the Panel on Euthanasia of the American Veterinary Medical Association. The protocol was approved by the Institutional Animal Care and Use Committee of the University at Albany (Protocol #07-010).

Cells were plated at 60,000 cells/well in non-adherent conditions in 6 well plates containing DMEM (Life Technologies), N2 (Life Technologies), B27 (Life Technologies), 2 mML-glutamine (Life Technologies), 1 mM sodium pyruvate, 1 mM N-Acetyl-L-Cysteine (Sigma), and 20 ng/ml FGF2 and EGF (Life Technologies). After overnight incubation at 35 °C in 5% CO_2_, cells were transduced in parallel with either the Bmi1 overexpression or control empty vector lentivirus, with an MOI of 10. (We found that adult NSCs survived transduction more reliably when allowed to recover overnight in this way; eNSCs did not require this recovery period.) Cells were grown in non-adherent conditions for 7d to form neurospheres and were fed fresh medium as above every other day. Cells were passaged every seven days by spinning down neurospheres, gently dissociating using papain ((Worthington) for 20 min at 37 °C with gentle agitation and replating at 60,000 cells/well. Neurospheres were harvested for RNA isolation at the end of week 4. Two replicate RNA-seq experiments performed using RNA isolated at the end of week 1 yielded similar expression changes as seen at the end of week 4 (Supplementary Fig. S3).

Preparation of embryonic (E18) NSCs was done similarly, but cells isolated from embryonic cortex were plated at 30,000 cells per well, were transduced acutely rather than after overnight incubation, and RNA was isolated at the end of week 3 (after two passages).

Proliferation of NSCs was measured by disaggregating cells at the end of first or second passage and counting. Colony size was quantified using Cellprofiler^41^. Production of neurons from NSCs was measured by plating cells into poly-l-lysine-coated six-well plates (Costar) in serum-free culture medium for neurosphere growth but lacking EGF, and allowing cells to differentiate 14 days before fixing and stained with antibody to beta-tublin III (Sigma T8660) followed by goat anti-MsIgG2b-AF647 (Thermo Scientific). Image analysis of stained cells was performed using Cellprofiler^41^.

### RNA extraction and microarray hybridization

Neurospheres were spun down at 40 × g and collected. Total RNA was isolated using RNAeasy Plus Micro kit (Qiagen) and was converted to biotin-labeled cRNA according to the NuGEN Ovation RNA Amp System v2 protocol. After purification with Qiagen RNeasy spin columns and chemical fragmentation, the cRNAs were hybridized to Affymetrix mouse genome 430 2.0 microarrays. Hybridization, washing, and staining of the microarrays were done according to Affymetrix standard protocols on a FS450 system. The arrays were scanned using an Affymetrix Gene Chip 30007 G Scanner, and the images were converted to expression values using the Affymetrix GeneChip Command Console software. Four biological replicates were hybridized for each sample. For qPCR analysis, cDNA was prepared using a cDNA synthesis kit (USB) and the resulting cDNA quantified using SYBR green chemistry on an ABI 7500 Fast real time PCR system. A housekeeping gene was used for normalization (GAPDH). The primers used in the qPCR are listed in Supplementary Table S6.

### High throughput RNA sequencing (RNA-seq)

To prepare samples for Illumina sequencing, 150–250 ng of RNA prepared from aNSCs or eNSCs (using the RNAeasy Plus Micro kit; Qiagen) was first treated to remove ribosomal RNA using the Ribo-Zero Magnetic Kit (Epicentre, Madison, WI), using the protocol for low input samples. Sequencing libraries were prepared using the ScriptSeq V2 RNA-seq Kit (Epicentre), again using the low input protocol. Samples were bar-coded (2–4 samples per lane) and sequenced on an Illumina Hi-Seq. 2500 at the University at Buffalo Next-Generation Sequencing and Expression Analysis Core.

### Data Analysis

Microarray CEL files were processed using the Cistrome web tool^22^. The four replicates of each condition were averaged and used to calculate log_2_ changes in expression with accompanying p-values (paired t-test) and false discovery rates^42^. For RNA-seq, reads were aligned to the genome (mm9 genome build) and transcripts assigned using Tophat, Cufflinks, and Cuffdiff at the Galaxy server^36,43^. Further analysis was performed using Excel. Clustering was performed using Cluster 3.0 (http://bonsai.ims.utokyo.ac.jp/Bmdehoon/software/cluster/) and visualized using Java Treeview (http://jtreeview.sourceforge.net/)^44^. Gene ontology analysis was performed using the Generic Gene Ontology Term Finder^45^. For comparison with other data sets, data was downloaded or obtained after personal communication (Yukiko Gotoh)^46^ and processed at the Cistrome website^22^.

### Data availability

Microarray and RNA-seq data from this study have been deposited at the Arrayexpress repository (E-MTAB-3419 for microarray data and E-MTAB-3491 for sequence data). Processed data is available as Supplementary Data Files 1–3.

## Electronic supplementary material

Supplementary Information

Supplementary Table S2

Supplementary Table S3

Supplementary Table S4

Supplementary Table S5

Dataset 1

Dataset 2

Dataset 3
