# Supplementary material for: Effect of Bmi1 over-expression on gene expression in adult and embryonic murine neural stem cells
Source: Sci Rep. 2018 May 10;8:7464. doi: 10.1038/s41598-018-25921-8 (PMC5945652; doi:10.1038/s41598-018-25921-8)
Supplement: Supplementary file 1 — Supplementary Information [file 41598_2018_25921_MOESM1_ESM.pdf]

# Supplementary Information

## Effect of Bmi1 over-expression on gene expression in adult and embryonic murine neural stem cells

Mythily Ganapathi[1], Nathan C. Boles[2], Carol Charniga[2], Steven Lotz[2], Melissa Campbell[2], Sally Temple[2], and Randall H. Morse[1,3]

[1]Laboratory of Molecular Genetics, Wadsworth Center, New York State Dept. of Health, Albany, NY, USA

[2]Neural Stem Cell Institute, Rensselaer, NY, USA

[3]Department of Biomedical Science, University at Albany School of Public Health, Albany, NY, USA

**Supplementary Figure S1. Enhancement of eNSC and aNSC self-renewal by Bmi1 overexpression.** Representative micrographs of (A) eNSCs and (B) aNSCs transduced with empty vector (control) or Bmi1 overexpression vector (Bmi1 oe) at 15 days (end of second passage) or 21/22 days (end of third passage) showing enhanced neurosphere formation in Bmi1 overexpressing NSCs. (C) Ratio of cell number, obtained by disaggregating neurospheres at the end of first and second passage, for equal numbers of eNSCs transfected with the Bmi1-overexpressing vector (Bmi1 oe) relative to cells transfected with the control vector. (D) Size of colonies derived from aNSCs after 1$^{st}$ passage measured with Cellprofiler software; n= number of colonies quantified. (E) BMI1 overexpression increases neuronal output. BMI1 overexpressing NPCs and control NPCs were plated and allowed to spontaneously differentiate. Cells were fixed and stained with antibody to beta-tubulin (Tubb3) and image analysis was carried out with Cellprofiler. (Left) The actual counts of neurons and total cells for each well and

condition is shown. BMI1 OE cells and H1 control cells showed similar numbers of total cells, but BMI1 had an increased number of neurons. (Right) The percentage of neurons for each well is shown.

**Supplementary Figure S2. Validation of microarray results by qPCR.** Reverse transcription followed by quantitative PCR was used to assess expression of genes up- or down-regulated in Bmi1 overexpressing aNSCs compared to control aNSCs. RNA from the same samples used for microarray analysis (Batch 1-4) as well as four additional biological replicates (Batch 5-7 and 9) were assessed and compared to results from four replicate microarray experiments. Error bars reflect standard deviations (n=4); N.D. means not determined. Data is shown for all genes that were analyzed by qPCR. The * for *Plp2* indicates that two probes on the microarray yielded different values for expression; Cistrome (used for processing microarray data; see Methods) did not produce an expression value for *Plp2*. The values for the two probes, obtained using Genespring (n=3) are shown instead.

**Supplementary Figure S3. Comparison of change in gene expression in *Bmi1*-overexpressing and control cells one week and four weeks following isolation and lentiviral transduction.** Only genes showing more than two-fold change in at least one of the data sets and having RPKM > 1.0 are shown.

**Supplementary Figure S4. Transcript levels and expression changes in aNSCs and eNSCs upon Bmi1 overexpression for genes mapping to GO categories of neurogenesis and cell adhesion.** Transcript levels (upper part of graphs, in reads per kilobase per million

mapped reads, RPKM) and $log_2$ change in expression upon *Bmi1* overexpression (lower part of graphs) for genes in the GO categories (A) “Neurogenesis” and (B) “Cell adhesion” that are down-regulated in either aNSCs or eNSCs. Graphs are divided according to RPKM level for clarity.

**Supplementary Figure S5. Comparison of gene expression changes in embryonic and adult NSCs caused by Bmi1 overexpression.** Genes having expression altered at least two-fold by Bmi1 overexpression in either eNSCs or aNSCs, and having stronger expression in one cell type over the other by at least two-fold, as indicated, are plotted for the two cell types.

**Supplementary Table S1. Bmi1 expression in replicate experiments.** RNA employed for each of four microarray experiments was used to prepare cDNA, which was subjected to qPCR analysis. SYBR green detection method based qPCR was done and GAPDH was used to quantitate the relative expression. Three replicates were used for each sample.

**Supplementary Table S2. Gene ontology analysis of genes down-regulated at least two fold in aNSCs by Bmi1 overexpression.**

**Supplementary Table S3. Gene ontology analysisis of genes up-regulated at least two fold in aNSCs by Bmi1 overexpression.**

**Supplementary Table S4. Effect of Bmi1 overexpression on expression of 84 imprinted genes.**

**Supplementary Table S5. Genes belonging to clusters shown in Fig. 2.**

**Supplementary Table S6. Primers used in this study.**

**Supplementary Data File 1. Microarray data for four replicate experiments measuring gene expression in aNSCs transduced with control empty vector or Bmi1 overexpression vector.** Expression values represent log2 of intensities.

**Supplementary Data File 2. Gene expression from RNA-seq for eNSCs transduced with control empty vector or Bmi1 overexpression vector.** Values are averages from two replicate experiments.

**Supplementary Data File 3. Gene expression from RNA-seq for aNSCs transduced with control empty vector or Bmi1 overexpression vector.** Values are averages from two replicate experiments.

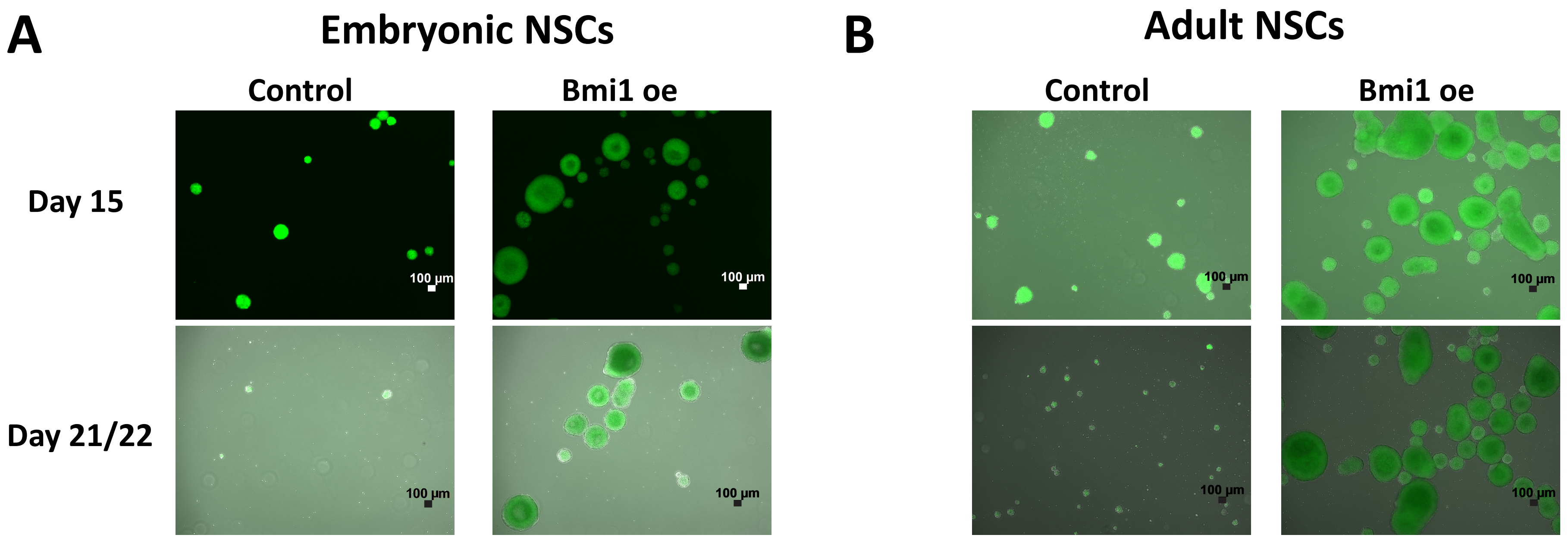


C

| Bmi1 oe/control growth | First passage | Second passage |
|---|---|---|
| Expt 1 | 1.1 | 7.5 |
| Expt 2 | 2.7 | 120 |

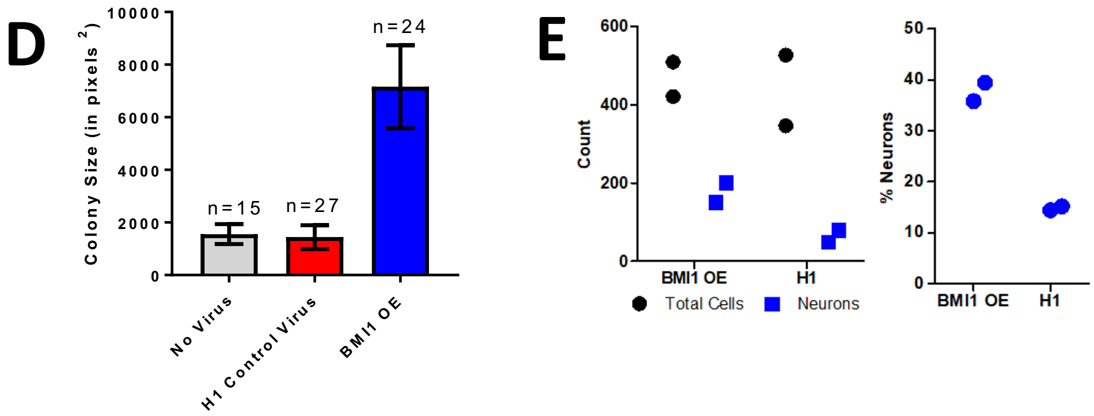


**Supplementary Figure S1. Enhancement of eNSC and aNSC self-renewal by Bmi1 overexpression.** Representative micrographs of (A) eNSCs and (B) aNSCs transduced with empty vector (control) or Bmi1 overexpression vector (Bmi1 oe) at 15 days (end of second passage) or 21/22 days (end of third passage) showing enhanced neurosphere formation in Bmi1 overexpressing NSCs. (C) Ratio of cell number, obtained by disaggregating neurospheres at the end of first and second passage, for equal numbers of eNSCs transfected with the Bmi1-overexpressing vector (Bmi1 oe) relative to cells transfected with the control vector. (D) Size of colonies derived from aNSCs after 1$^{st}$ passage measured with Cellprofiler software; n= number of colonies quantified. (E) BMI1 overexpression increases neuronal output. BMI1 overexpressing NPCs and control NPCs were plated and allowed to spontaneously differentiate. Cells were fixed and stained with antibody to beta-tubulin (Tubb3) and image analysis was carried out with Cellprofiler. (Left) The actual counts of neurons and total cells for each well and condition is shown. BMI1 OE cells and H1 control cells showed similar numbers of total cells, but BMI1 had an increased number of neurons. (Right) The percentage of neurons for each well is shown.

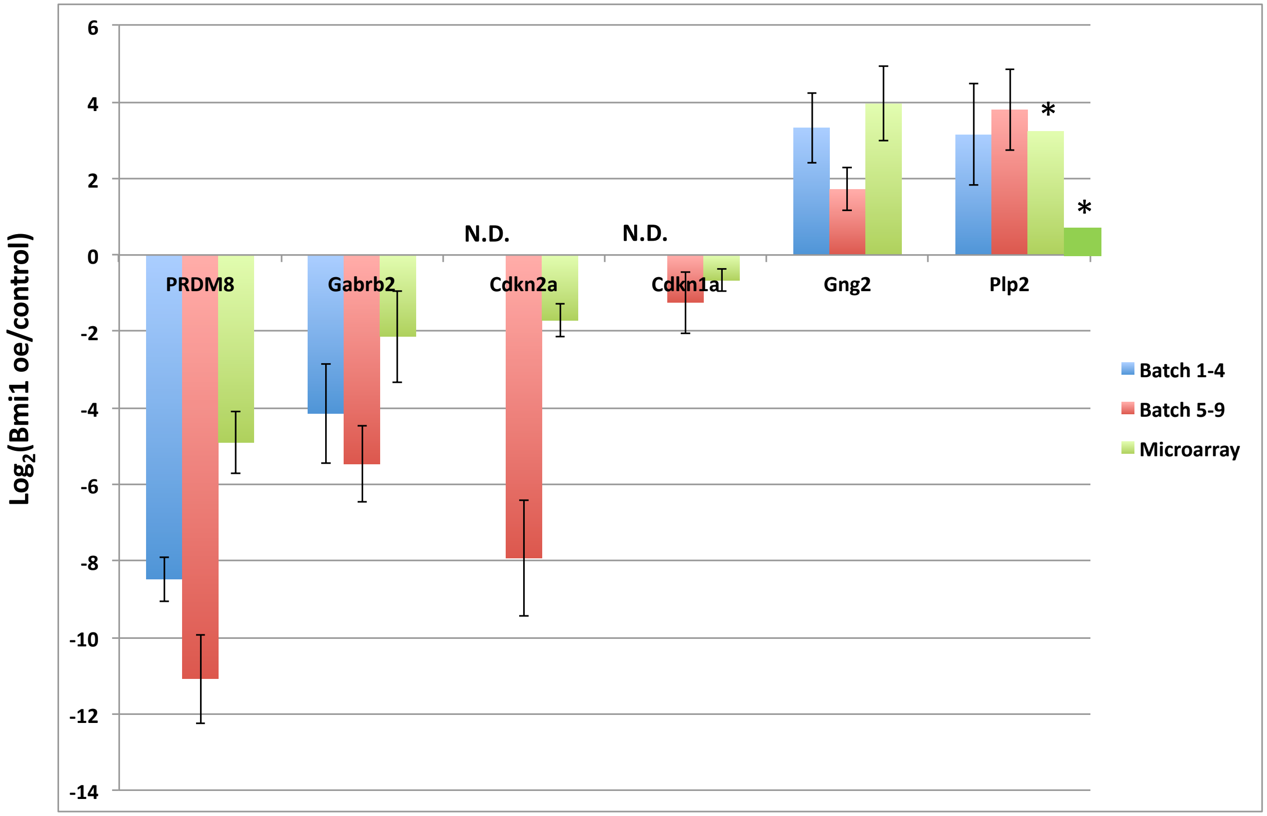


**Supplementary Figure S2. Validation of microarray results by qPCR.** Reverse transcription followed by quantitative PCR was used to assess expression of genes up- or down-regulated in Bmi1 overexpressing aNSCs compared to control aNSCs. RNA from the same samples used for microarray analysis (Batch 1-4) as well as four additional biological replicates (Batch 5-7 and 9) were assessed and compared to results from four replicate microarray experiments. Error bars reflect standard deviations (n=4); N.D. means not determined. Data is shown for all genes that were analyzed by qPCR. The * for *Plp2* indicates that two probes on the microarray yielded different values for expression; Cistrome (used for processing microarray data; see Methods) did not produce an expression value for *Plp2*. The values for the two probes, obtained using Genespring (n=3) are shown instead.

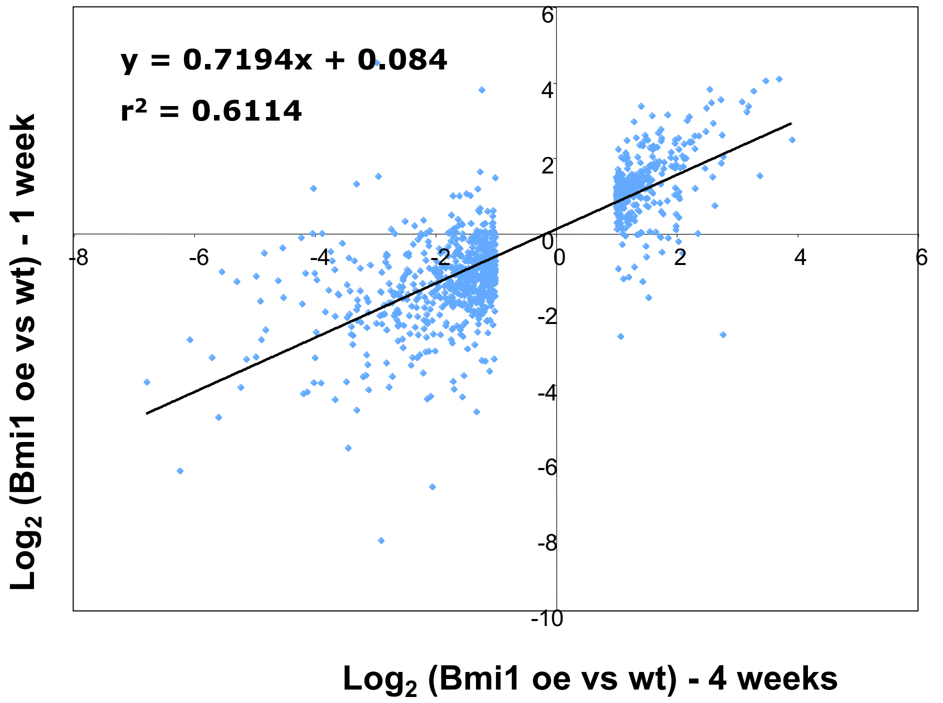


**Supplementary Figure S3. Comparison of change in gene expression in *Bmi1*-overexpressing and control cells one week and four weeks following isolation and lentiviral transduction.** Only genes showing more than two-fold change in at least one of the data sets and having RPKM > 1.0 are shown.

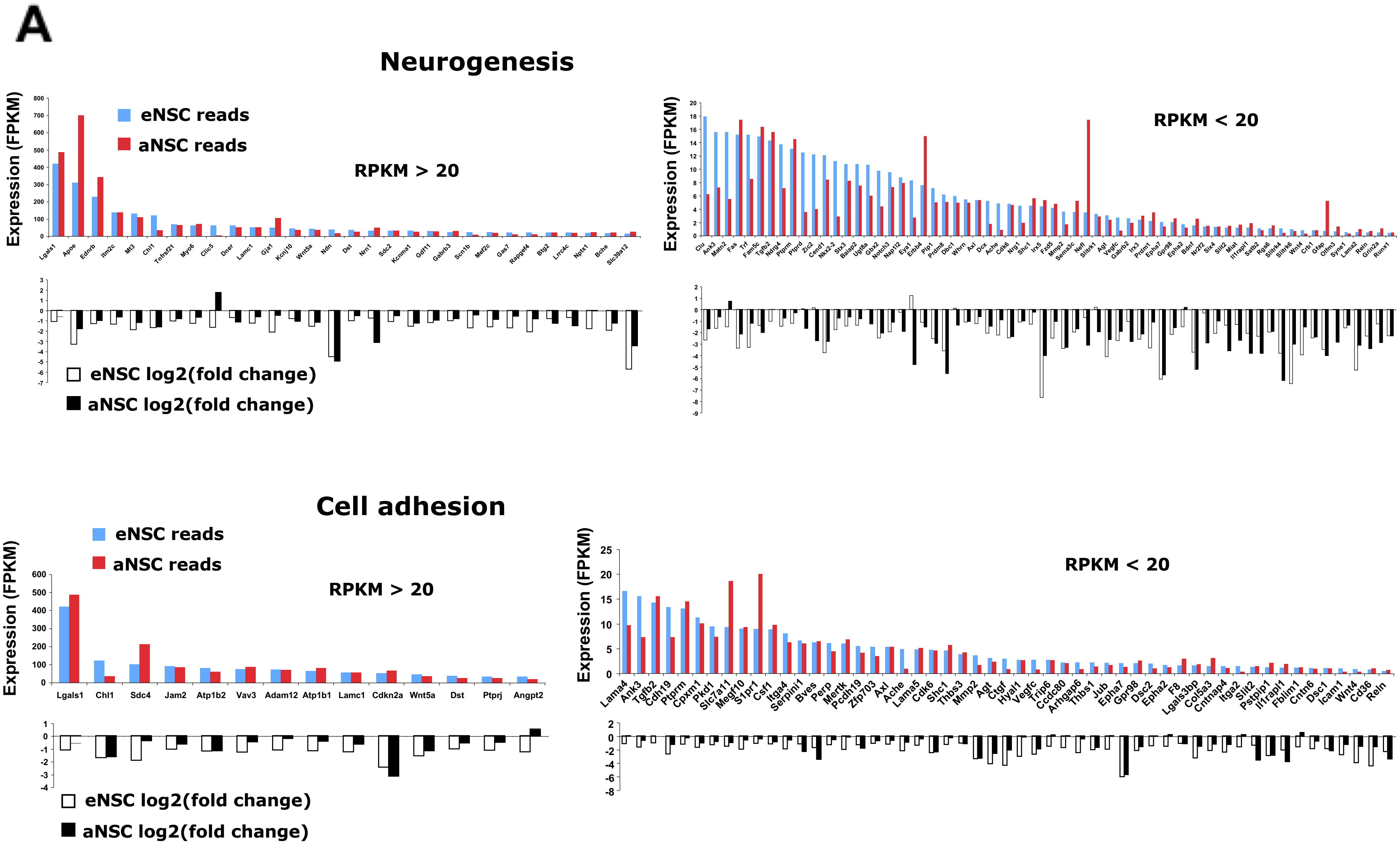


**Supplementary Figure S4. Transcript levels and expression changes in aNSCs and eNSCs upon Bmi1 overexpression for genes mapping to GO categories of neurogenesis and cell adhesion.** Transcript levels (upper part of graphs, in reads per kilobase per million mapped reads, RPKM) and $\log_2$ change in expression upon *Bmi1* overexpression (lower part of graphs) for genes in the GO categories (A) “Neurogenesis” and (B) “Cell adhesion” that are down-regulated in either aNSCs or eNSCs. Graphs are divided according to RPKM level for clarity.

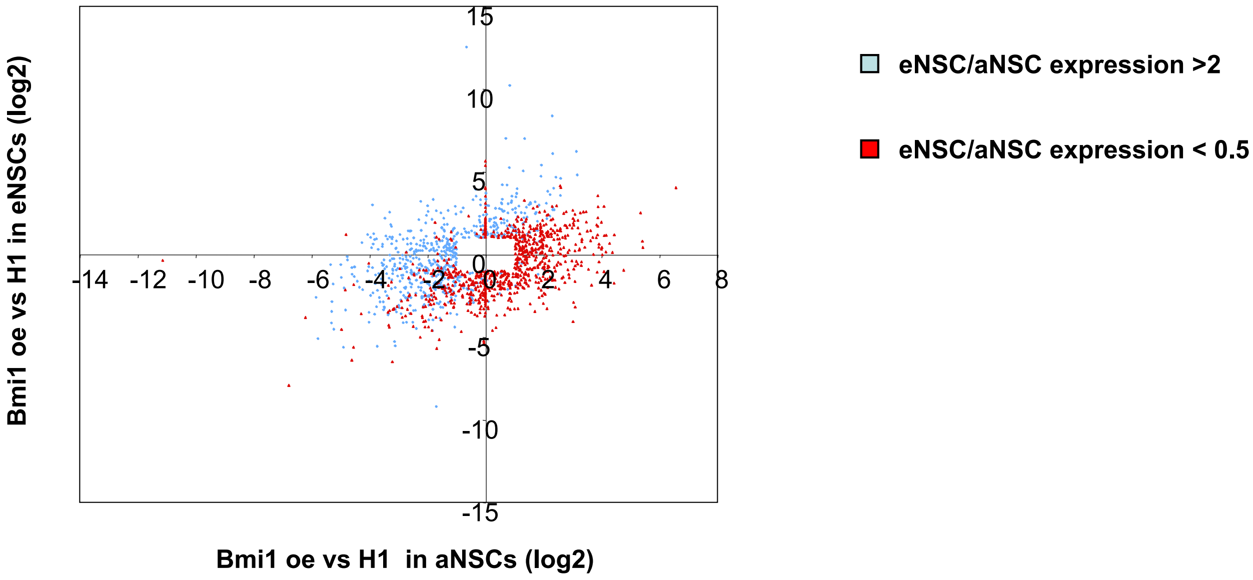


**Supplementary Figure S5. Comparison of gene expression changes in embryonic and adult NSCs caused by Bmi1 overexpression.** Genes having expression altered at least two-fold by Bmi1 overexpression in either eNSCs or aNSCs, and having stronger expression in one cell type over the other by at least two-fold, as indicated, are plotted for the two cell types.

| Microarray Sample | Bmi1 fold change in Bmi1 OE/Empty vector control |
|---|---|
| Replicate 1 | 23.65 |
| Replicate 2 | 18.58 |
| Replicate 3 | 14.97 |
| Replicate 4 | 3.94 |

**Supplementary Table S1:** RNA employed for each of four microarray experiments was used to prepare cDNA, which was subjected to qPCR analysis. SYBR green detection method based qPCR was done and GAPDH was used to quantitate the relative expression. Three replicates were used for each sample.

**Supplementary Table S6. Primers used in this study.**

| Primer | Sequence |
|---|---|
| Gabrb2_FP | ATGTCGCTGGTTAAAGAGACG |
| Gabrb2_RP | CTGCCACTCGGTTGTCCAAA |
| mPRDM8_FP | TTC TGA CAC CTT CCT TCC T |
| mPRDM8_RP | CAG ATC CTA AGC TCC TTC C |
| mArf_FP | AAA ACC CTC TCT TGG AGT GGG |
| mArf_RP | GCA GGT TCT TGG TCA CTG TGA G |
| mInk4a_FP | GAT GGA GCC CGG ACT ACA GAA G |
| mInk4a_RP | CTG TTT CAA CGC CCA GCT CTC |
| Bmi-1_FP | AAATCAGGGGGTTGAAAAATCT |
| Bmi-1_RP | GCTAACCACCAATCTTCCTTTG |
| p21_FP | TGACAGATTTCTATCACTCCAAGC |
| p21_RP | ACTTTAAGTTTGGAGACTGGGAGA |
| Plp2_FP | ATGGCGGATTCTGAGCGTC |
| Plp2_RP | GCACTGAAGCAAATCAAGATCAC |
| Gng2_FP | ACCGCCAGCATAGCACAAG |
| Gng2_RP | AGTAGGCCATCAAGTCAGCAG |
